# Early Spalling Analysis of Large Particles in High-Cr Steel during Thermal Fatigue: Relevant Mechanisms

**DOI:** 10.3390/ma15196705

**Published:** 2022-09-27

**Authors:** David Bombač, Goran Kugler, Jaka Burja, Milan Terčelj

**Affiliations:** 1 Department for Materials and Metallurgy, Faculty of Natural Sciences and Engineering, University of Ljubljana, 1000 Ljubljana, Slovenia; 2 Institute of Metals and Technology, 1000 Ljubljana, Slovenia

**Keywords:** thermal fatigue, surface degradation, large particle spalling, high-Cr steel, oxidation, crack growth

## Abstract

The aim of this study was to investigate the surface deterioration of high-Cr roll steel caused by the spalling of larger particles during thermal fatigue. The mechanisms of surface deterioration due to spalling of larger particles are discussed. Using a laboratory thermal fatigue test that replicates hot rolling conditions, samples were tested cyclically (up to 4500 times) at maximum cycle temperatures of 500, 600 and 700 °C, followed by water cooling. Specimens with surface deterioration were selected for analysis, revealing important influencing parameters, i.e., the combination of test temperatures, chemical composition, thermal stress and microstructural properties, leading to oxidation-assisted crack growth in different directions and consequent surface deterioration due to early spalling of larger particles. Here, we describe the mechanisms of crack propagation, especially in the lateral direction, and their relation to the subsequent spalling of larger particles, which depend on the influence of the local chemical composition on the microstructural constituents, as well as their distribution and properties. The results obtained in this study can be used in the development of roll steel microstructures with improved resistance to the identified mechanisms of surface degradation.

## 1. Introduction

Work rolls are subjected to high thermal, mechanical, tribological and chemical loads during hot rolling. These loads cause damage that manifests itself in the form of plastic deformation, cracks, particle spalling, oxidation and roll wear. These factors determine the service life of industrial rolls for hot forming. Work rolls are usually made from indefinite chill double pour (ICDP) cast iron, high-chromium iron, high-chromium steels and high-speed steels (HSS). In industrial operation, work rolls are usually heated to 500–700 °C at the contact surface and immediately cooled with water. The cycles of heating and cooling are essentially thermal cycles, leading to thermal fatigue. The material is exposed to constantly changing mechanical stresses and is subject to strong oxidation conditions. When the temperature increases to 500 °C or more, oxidation becomes an important factor.

The literature indicates that the oxidation rate of hot work tool steels becomes noticeable at 500 °C and increases rapidly at 700 °C [1]. However, there are differences in the oxidation of chromium hot work tool steels. The choice of a an appropriate heat treatment and the addition of molybdenum slow down the oxidation process [2]. In addition, molybdenum-rich carbides are resistant to oxidation and remain intact, whereas the surrounding matrix is completely oxidized [1,3]. The occurrence of spalling in the surface layer is considered critical, as both the surface quality of the rolled product and the ability of the rolls to resist fractures (critical crack size) are called into question. Considerable effort has been invested in the study of early roll surface spalling, and recent studies suggest that spalling is likely related to roll oxidation mechanisms [3,4,5,6,7,8,9,10,11,12,13]. The increase in roll surface roughness is generally due to coalescence of short internal and external cracks, as well as abrasion and adhesive wear. Laboratory tests confirmed that oxidation plays a role in abrasive wear, affecting not only crack propagation but also material removal. However, material removal occurs only in smaller and expected volume ranges [3,11,12,14,15,16,17,18]. Conversely, the unexpected early spalling of larger volumes of material leads to a significant change in roll surface topography and requires immediate roll replacement. In this context, industrial case studies that correlate spalling with microstructure are valuable. Colàs et al. [9] analyzed the microstructure of failed high-Cr rolls due to early spalling and concluded that oxidation with the formation of oxidation loops was significant. In addition, the study results indicated varying oxidation rates of microstructural components, with oxidation pathways observed and material strength decreased when two oxidized cracks joined to form a loop with a brittle oxidized layer encapsulating healthy material. Studies published in the literature to date consider either only laboratory oxidation in a humid atmosphere [4,5,6,10,11,13,15,19,20,21,22,23,24,25,26,27,28,29] or only thermal fatigue tests [3,18,30,31,32,33,34]. Although these studies are welcomed, the response of the material differs in the presence of both phenomena.

The process of crack formation and propagation associated with the increased spalling of large particles (over 300 µm) is not yet fully understood, in contrast to the spalling of smaller particles, which has been described in the literature. In this study, we discuss the mechanisms of degradation of high-chromium steel for work rolls due to early spalling of large particles from the surface. In order to investigate thermal fatigue simultaneously with oxidation leading to early spalling of larger particles, we employed a thermal fatigue test that can simulate both phenomena.

## 2. Materials and Methods

**Figure 1:**
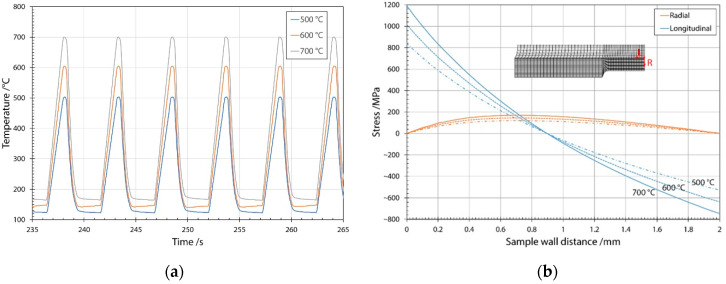
Temperatures measured during the thermal fatigue test with a Gleeble 1500D thermomechanical simulator and stresses determined on the specimen. (**a**) Cycle temperature measured during thermal cycling at maximum temperatures of 500, 600 and 700 °C; (**b**) thermal stresses as determined with FEM in radial (R) and lateral (L) directions and FEM mesh used with origin of distance.

In this investigation, we used a centrifugally cast roll made of a high-Cr (Cr12) steel; its chemical composition is presented in Table 1. Thermal fatigue borehole samples with an investigated diameter of 8 mm and wall thickness of 2 mm were machined out of the centrifugally cast roller shell and thermally fatigued on a Gleeble 1500D thermomechanical simulator (Dynamic Systems Inc., Poestenkill, NY, USA) for a finite number of cycles. Details of the test rig can be found in references [30,31]. The samples were analyzed after 200, 500, 1000, 2500 and 4500 thermal cycles, with the maximum temperature in each thermal fatigue cycle reaching 500, 600 or 700 °C (see Figure 1a).

Borehole samples were heated conductively, cooled with water through the borehole and dried with compressed air before being heated in the next cycle. The test was computer-controlled with repeatable conditions in each cycle mimicking the conditions in the work rolls during hot rolling. Qualitative evaluation of thermal stresses was carried out using finite element method (FEM) in previous work [3], and Figure 1b shows a summary of the results. The maximum values of thermal stresses reached 170, 145 and 120 MPa in radial direction and 1190, 1016 and 840 MPa in the lateral (longitudinal) direction on the cooled surface at temperatures of 700, 600 and 500 °C, respectively. The results of the FEM analysis confirm the preferential direction of crack growth in the radial direction, with limited growth in the lateral direction. Crack growth in the radial direction is favored by the cracking of carbides perpendicular to the maximum thermal stresses.

To investigate the oxidation behavior, several specially prepared samples with polished surfaces were fabricated and exposed to air at room temperature for four months. The oxidation resistance of the surface microstructure (carbides and matrix) was expected to differ depending on the chemical composition, as well as an additional confirmation of the results from the thermal fatigue tests.

Light optical microscopy (Carl Zeiss AXIO Imager.A1m, Carl Zeiss QEC GmbH, Köln, Germany) and scanning electron microscopy (SEM) (JEOL 5610, JEOL ltd., Tokyo, Japan, and FEI NanoSEM, ThermoFisher Scientific, Waltham, MA, USA) were used to observe the surface (topology and cracks) and microstructure (oxidation and carbides). Chemical mapping of the microstructures was performed using energy-dispersive X-ray spectroscopy (EDX) coupled with SEM. Samples for light optical microscopy were etched with Murakami reagent to qualitatively visualize the distribution of chemical elements in the primary and eutectic carbides.

## 3. Results

### 3.1. Initial Microstructure in Connection to Oxidation

**Figure 2:**
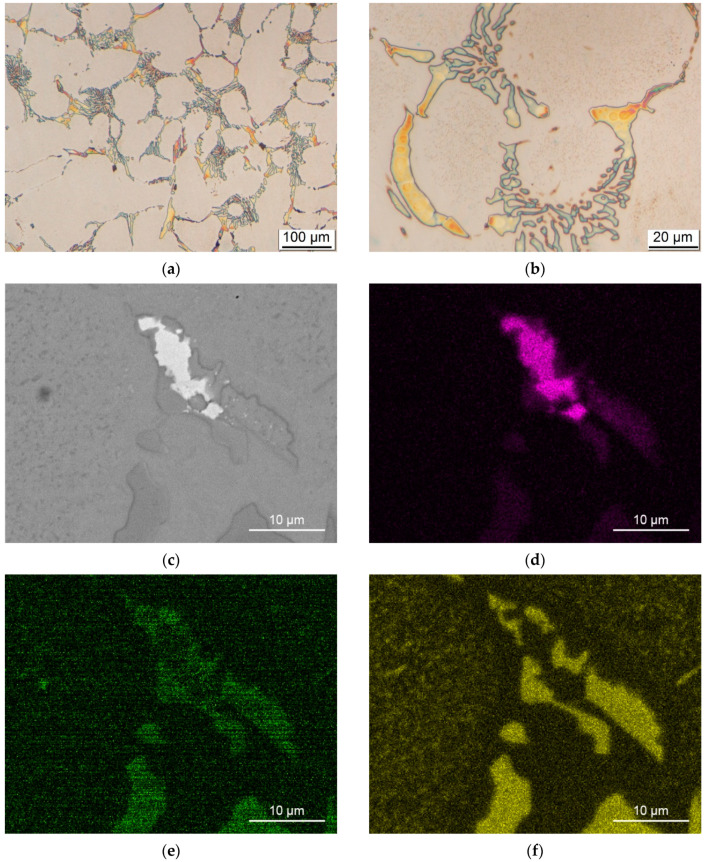
Initial microstructure of high-Cr roll steel. (**a**) Light optical micrograph of the starting material etched with Murakami etchant; (**b**) higher magnification micrograph of carbides; (**c**) SEM micrograph of the chemical mapping area; (**d**) map of Mo distribution in primary and eutectic carbides; (**e**) map of oxygen distribution showing oxidation of the surface layer in air for four months; and (**f**) map of Cr distribution in primary and eutectic carbides.

The microstructure of high-Cr steel from centrifugally cast roller steel consists of primary, eutectic and secondary carbides embedded in a martensitic matrix. Light optical micrographs showing the typical microstructure of high-Cr steel are shown in Figure 2. A detailed description of the microstructure of the same material can be found in references [3,30]. Quantitative metallography of the high-Cr roller material revealed 15.46 vol% M_7_C_3_ and 1.41 vol% M_2_C carbide, with an average measured hardness of 601 ± 10 HV_10_.

In our previous study [3], we showed a significant increase in oxidation in Cr- and Mo-poor regions, whereas Mo- and Cr-rich regions exhibited better oxidation resistance. These results indicate the importance of Mo content in carbides with respect to their oxidation kinetics and show the influence of Mo on crack growth and oxidation pathways. A similar observation was made by Delaunois et al. [35] with HSS rolls, who reported that oxidation had no effect on the Mo-rich carbides. Using Murakami solution as the etchant, the Mo distribution in the photomicrographs is qualitatively shown in Figure 2a,b, with the Mo-rich regions appearing as orange to brown and the Mo-poor regions appearing bluish. EDX analysis showed large chemical segregation of Mo, from 3–8 wt% in primary carbides and 1.9–2.9 wt% in eutectic carbides. Chemical segregation of alloying elements in as-cast rolls is common as a result of non-equilibrium conditions during solidification [36]. Figure 2c shows a SEM micrograph of a typical carbide in which the Mo-rich region in carbide is bright. The obtained elemental maps of Mo, O and Cr are depicted in Figure 2d–f, respectively. Prior to chemical mapping analysis, the sample was stored in air at room temperature for four months. More oxidation was observed in areas with lower Mo content. These results indicate the importance of Mo content in carbides in relation to their oxidation kinetics and show the influence of Mo on crack growth and oxidation pathways.

### 3.2. The Importance of Testing at High Temperatures

Testing at the maximum temperature of the thermal cycle of 500 °C did not result in significant deterioration of the surface of the specimen. The results of thermal fatigue of the same steel at a maximum temperature of 500 °C are discussed in our previous work [3,30]. In this work, which deals with the spalling mechanisms of large particles, only specimens thermally cycled at 600 and 700 °C provided significant results that warrant further investigation and discussion.

### 3.3. Spalling of Smaller Particles from Cooled Surface

**Figure 3:**
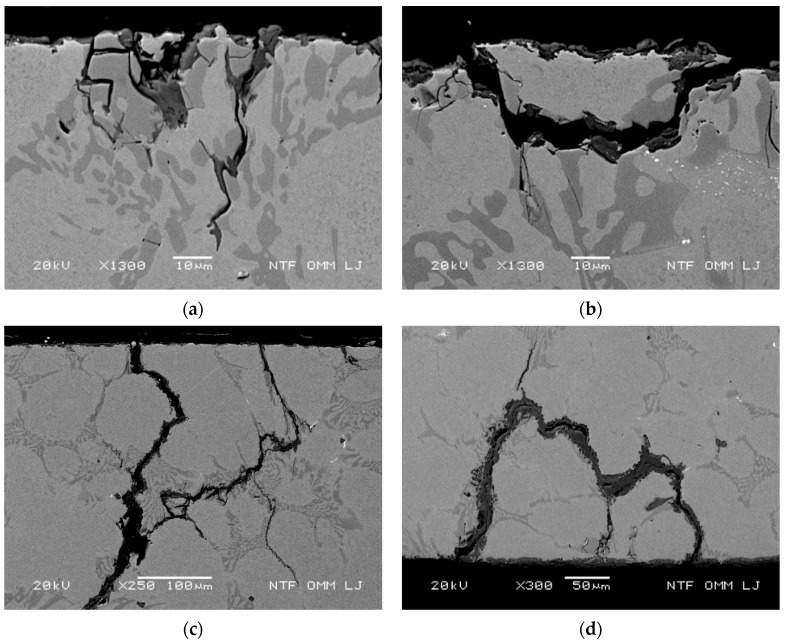
Mechanisms of removal of smaller particles leading to increased surface roughness. (**a**) Cracking and crushing of the enlarged carbide assembly at a maximum temperature of 700 °C and 200 thermal cycles; (**b**) spallation of smaller particles enclosed by carbides at 700 °C after 200 thermal cycles; (**c**) spallation of smaller particles by crack coalescence with the crack formed on the cooled surface at 700 °C and 200 thermal cycles; and (**d**) spallation of particles by strongly progressive oxidation at 700 °C and 1000 thermal cycles.

The process of initial spalling of small particles (100–300 µm in diameter) begins with crushing of the carbides. This is shown in Figure 3, where the material was thermally fatigued for 200 cycles at a maximum test temperature of 700 °C. The process involves crushing of the primary and eutectic carbides, as shown in Figure 3a, and removal of small parts of the matrix surrounded by cracked thick primary and eutectic carbides (see Figure 3b). In addition, the slightly elevated part (island with a length of 100–200 µm) shown in the middle of the micrograph in Figure 3b is related to the connection of two cracks formed on the cooled surface and propagating along the carbides, as shown in more detail in Figure 3c. Figure 3d also shows enhanced oxidation of the eutectic carbide. The process of entrapment of matrix material with connected oxidized cracks along the carbides shown in Figure 3d is thermally fatigued at 700 °C for 1000 cycles. The process is similar to that shown in Figure 3b, but the entrapped island is larger and surrounded by continuously arranged eutectic carbides with a distinct oxidation region along the cracks.

These processes increase the roughness of the rolls and are the main cause of the continuous increase in surface roughness during hot rolling. The mechanism leading to the increased surface roughness shown in Figure 3 occurred at a maximum temperature of 700 °C. Similar processes were also observed at lower test temperatures, with the difference being that the oxidation kinetics are slower, and a higher number of thermal cycles is needed for particle spalling to occur. As shown, oxidation of continuously arranged primary and eutectic carbide elements surrounding a matrix to form matrix islands is a transition process to remove enlarged surface particles (spalling).

### 3.4. Basic Conditions for Early Spalling of Larger Surface Particles

When primary and eutectic carbides are arranged in a row, accelerated radial crack growth can occur in both the radial and lateral directions. In general, early spalling of large particles (with a diameter greater than 300 µm) occurs due to the connection of two or more cracks deeper from the cooled surface, especially in cracks with accelerated crack growth. Accelerated crack growth in the radial direction, in combination with additional crack growth in the lateral direction (crack branching) in area deeper from the cooled surface, is the basis for early spalling of large particles.

#### 3.4.1. Thermal Stresses, Pronounced Carbide Cracking and Oxidation-Aided Crack Growth of Radially Growing Cracks

The thermal stresses in the lateral direction of the specimen (i.e., lateral direction in the sample axial cross section) cause crack growth in the radial direction. The maximum thermal stress in the lateral direction at a maximum thermal cycle temperature of 700 °C was found to be about 1190 MPa on the cooled surface and −750 MPa on the outer wall of specimen. The thermal stress in the radial direction, which is responsible for crack growth in the lateral direction, is much lower compared to the stress in the lateral direction at the same test temperature and was found to be 170 MPa at a depth of 700 µm [3]. Cracking in carbides (eutectics) is a result of thermal stresses caused by the temperature gradient in the specimen and stresses caused by an increased volume of oxidized material near the carbides; this also accelerates the growth of cracks in the lateral direction. Due to the increased axial (lateral) thermal stresses, the density and length of radial cracks, i.e., cracks perpendicular to the axial thermal stresses, are also increased compared to the same parameters in lateral crack directions.

**Figure 4:**
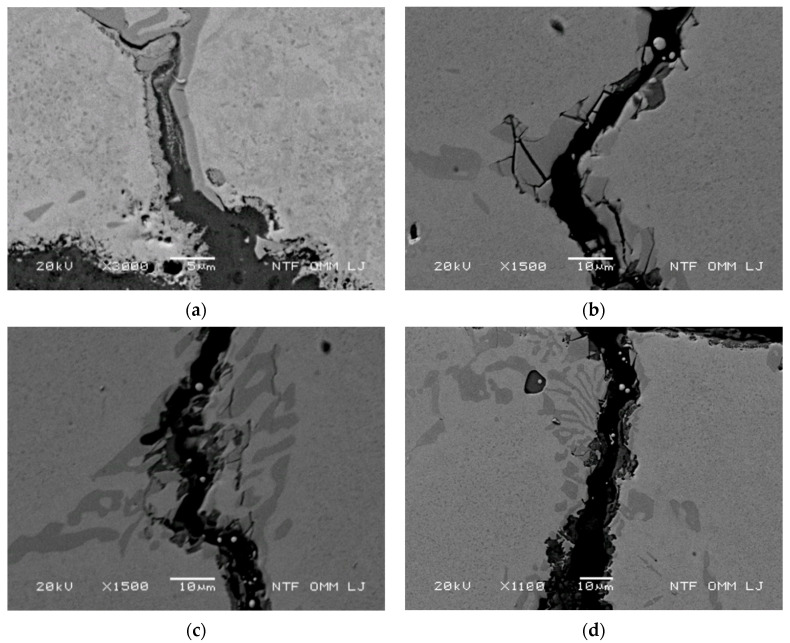
Main mechanism for accelerated growth of cracks in the radial direction at 700 °C after 2500 thermal cycles. (**a**) Oxidation of a narrow band adjacent to primary carbides; (**b**) cracking and oxidation of primary carbides; (**c**) initial stage and (**d**) later stage carbide cracking and oxidation of eutectic carbides with sites for EDX analysis.

The increased oxidation is related to the reduced Cr content in the matrix in the narrow band along the carbides (primary and eutectic) and the matrix, where many small eutectic carbides are present in the cluster, as well as to the cracking of the primary and eutectic carbides. The Cr content in the matrix along the narrow band along the primary carbides, as well as in the matrix surrounding the eutectic carbide clusters, was found to be lower than the Cr content elsewhere in the matrix. The Cr content measured with EDX was in the range of 4–5 wt% in the depleted areas, whereas 5.5–8.5 wt% Cr content was measured elsewhere in the matrix. In the Cr-depleted regions, accelerated oxidation of the narrow bands adjacent to the carbides was observed [3], with oxidation-aided crack growth occurring in the direction of the successive carbides, as shown in Figure 4a. In addition, cracking of primary and eutectic carbides also occurred in these areas, as shown in Figure 4b. The behavior shown in Figure 4a, i.e., oxidation in a narrow band along the carbides, is characteristic of lower thermal stresses (in areas deeper from the cooled surface). The behavior shown in Figure 4b, i.e., cracking of carbides, is characteristic of higher thermal stresses. The initial cracking of the eutectic carbides and oxidation of the eutectic carbides are also shown in Figure 4c, whereas the later stage of these processes (initial cracking following the pronounced oxidation of eutectic carbide) is shown in Figure 4d. Thus, the Cr-depleted bands along the carbides in deeper areas allow crack growth to change from predominant radial (i.e., in areas of increased thermal stress) to oblique or lateral, as lower thermal stresses prevail in areas deeper from the cooled surface. This also allows lateral cracks to connect to the radial cracks and consequently allows for early spalling of larger particles.

#### 3.4.2. Conditions Causing Crack Growth Direction Change from Radial to Lateral

In general, longitudinal thermal stresses initially cause crack growth in the radial direction, approximately perpendicular to the maximum thermal stress following carbide arrangement (carbide pathway). When the carbide arrangement in the radial direction is interrupted, a change in the crack growth direction occurs. Deeper from the cooled surface, the thermal stresses are much lower, and consequently, the cracking of carbides is reduced. Oxidation-aided crack growth occurs in the narrow band adjacent to the carbides, where lower Cr content was observed.

**Figure 5:**
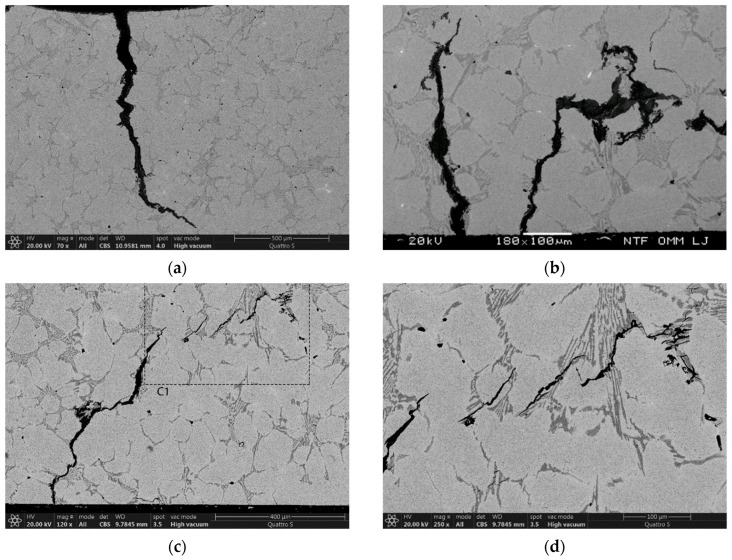

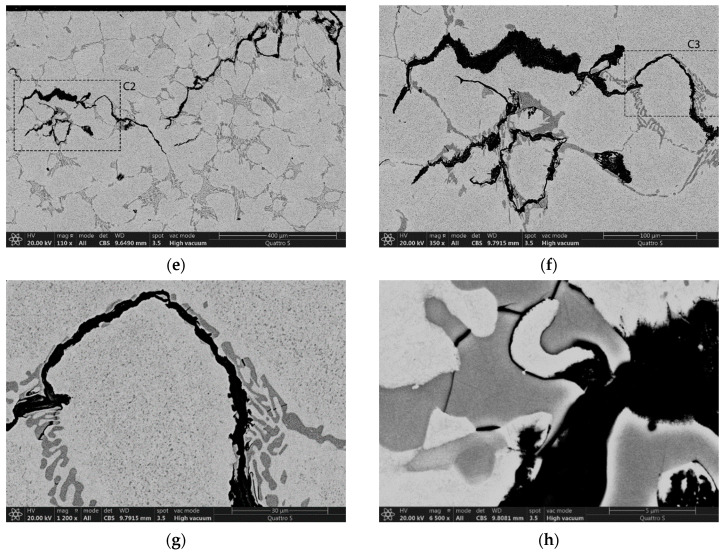
Crack growth change from the radial to lateral direction at 700 °C. (**a**) Crack growth deviation from radial direction to complex oblique direction due to reduced thermal stresses deeper in the specimen after 4500 thermal cycles; (**b**) growth direction change due to interruption of successive carbide assembly in the radial direction (crack on the right side) after 2500 thermal cycles; (**c**) crack growth direction change from oblique to lateral direction after 4500 thermal cycles with marked area for detail C1; (**d**) detail C1 with cracking of carbides in front of the crack tip; (**e**) crack growth direction change from oblique to simple in the lateral direction after 4500 thermal cycles and marked area for detail C2; (**f**) detail C2 with oxidation and cracking of carbides in the lateral direction and marked area of detail C3; (**g**) detail C3 with oxidation in the narrow band adjacent to carbides; and (**h**) oxidation and cracking of eutectic carbides after 4500 thermal cycles.

For example, in Figure 5a, in later stage of the crack growth, the direction of crack growth changes from the radial to the lateral (oblique) direction. Figure 5b shows two radially growing cracks, where the left crack reaches a length of about 700 µm, whereas the right crack kinks at a depth of about 400 µm and starts growing in the lateral direction. This occurs as a result of a break in the successive arrangement of carbides in the radial direction. A deviation in crack growth to the right (i.e., in the lateral direction) is observed, where a continued successive arrangement of carbides is present. Moreover, continuation of the carbide arrangement in the lateral direction due to oxidation leads to increased thermal stresses at the crack tip (i.e., also in the lateral direction) and bending stresses that break the carbides. At the point where the direction of crack growth changes, a slightly thicker oxidized region of the crack channel is visible, which further increases the stress around the oxidized region due to the increased volume of oxidized material. This promotes crack growth in the lateral direction. Because the matrix material is less brittle than carbides, continuation of the crack in the radial direction is hindered. If the orientation of the crack growth is not perpendicular (inclination angle of about 60°) to the cooled surface, further approach to the lateral direction can be achieved earlier if the carbides are arranged in a suitable order, as shown in Figure 5c, with detail C1 in Figure 5d. In such cases (i.e., when the crack growth deviates from the vertical direction), the stress field at the crack tip is also rotated, which consequently causes cracking of the precipitated carbides in front of the crack tip approximately in the direction of the crack inclination angle. Similarly, the shift in crack growth from the direction with an inclination angle of about 45° to the lateral direction is also shown in Figure 5e (see also the marked points for detail C2). Figure 5f shows a detail (C2) for the point marked in Figure 5e, where both cracking and oxidation of carbides, both oriented in the lateral direction, are visible. Oxidation follows the Cr-depleted path along the carbides despite the very challenging shape of the carbides (shown in Figure 5g on the left side). The cracking of eutectic carbides, in addition to oxidation, is shown in Figure 5h.

#### 3.4.3. Role of Larger Eutectic Carbide Assembly in Determining Crack Growth Direction

**Figure 6:**
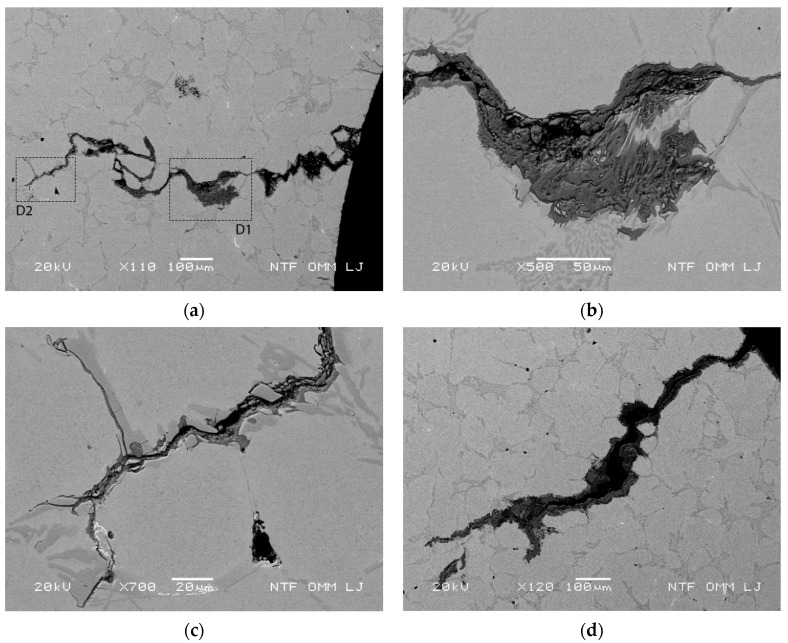
Crack growth and oxidation of crack channel in the path of a continuous and successive arrangement of eutectic carbide assembly in different directions. (**a**) Growth in the radial direction at an inclination angle of 70° in the pathway of several successively arranged enlarged eutectics with marked areas for detail regions D1 and D2 at 700 °C after 1000 thermal cycles; (**b**) micrograph of detail region D1 with initial crack branching due to eutectic oxidation in the lateral direction at a depth of about 500 μm; (**c**) micrograph of detail area D2 with crack growth direction change along primary carbides in the Cr-depleted band and eutectic carbides due to reduced thermal stress at a depth of about 1000 μm; and (**d**) simultaneous crack growth in the radial and lateral directions at 600 °C after 1000 thermal cycles.

Eutectic and primary carbides arranged in a continuous and successive manner in the lateral direction allow for crack growth due to oxidation and cracking of the carbides. Crack formation at a test temperature of 600 °C and 1000 thermal cycles, i.e., at a relatively low temperature and early stage of testing, is shown in Figure 6. The crack shown in Figure 6a (see also the marked areas for details D1 and D2) grows at an inclination angle of about 70° with respect to the cooled surface, as there were no successive carbides in the purely vertical (perpendicular to the cooled surface) direction. Furthermore, Figure 6b shows typical details (area of detail D1 in Figure 6a) of the third enlarged oxidized regions of the eutectic carbides to a depth of about 500 µm. Oxidation of the eutectic regions in the lateral direction is not yet complete. Thus, further crack growth begins in the lateral direction, and oxide growth continues in the next stage in the lateral direction. The chemical composition was analyzed with EDX for the eutectic carbides and the matrix near the eutectic carbides. The EDX results show that the Mo content in both domains is lower compared to the other eutectic domains. The measured Mo values are 0.50–0.60 wt% for the matrix and 1.8–2.1 wt% for the eutectic carbides, where an accelerated oxidation rate was observed. As mentioned above, increased oxidized eutectic domains oriented in the lateral direction increase the stresses in the surrounding areas, leading to accelerated crack formation and oxidation in the direction of the sequential arrangement of the primary and eutectic carbides, i.e., to the growth of cracks in the lateral direction. In the last part of the crack (at a depth of about 1000 µm), an additional deviation from the vertical direction can be observed in Figure 6c (see marked location for detail D2 in Figure 6a). This deviation occurs due to reduced thermal stresses, as the cracks start to grow in both directions at an inclination angle of about 45°, i.e., they start to follow carbide pathways that deviate significantly from the vertical direction. Thus, from all three points mentioned above (see Figure 6c), the cracks can grow simultaneously in the lateral direction in a further step. Moreover, even in the case of the considered enlarged eutectics (Figure 6a and detail D1 (Figure 6c)), the linkage with the next radial crack would lead to spalling of particles with smaller (thinner) size. Figure 6d shows lateral crack growth with enlarged eutectics. Further crack growth in the lateral direction can thus lead to linkage with nearby radially growing cracks.

#### 3.4.4. Connection of Oxidized Regions in the Lateral Direction between Primary Cracks Growing in the Radial Direction

**Figure 7:**
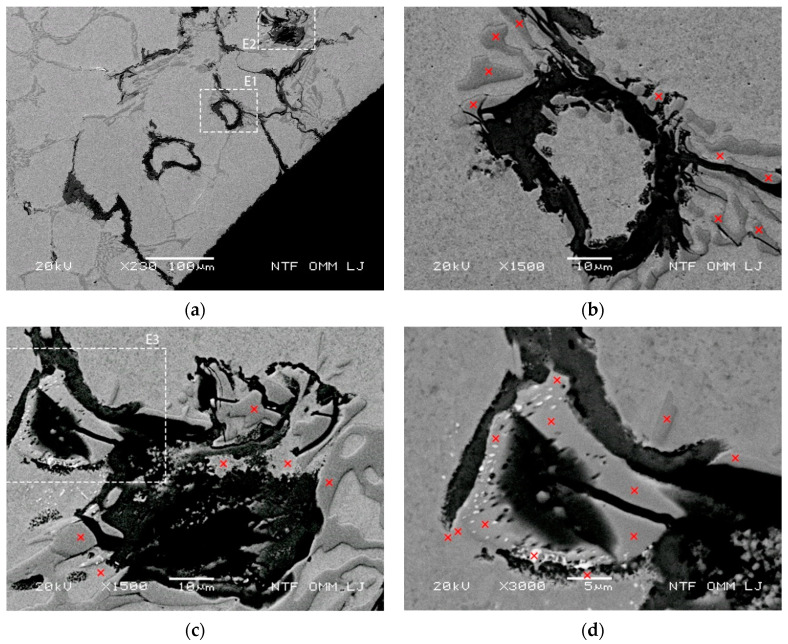
Regions of reduced Mo content in eutectic carbide with early carbide cracking and oxidation. (**a**) Micrograph of cracking and oxidation at 700 °C after 200 thermal cycles with marked areas for details E1 and E2; (**b**) micrograph of oxidation loop from area E1 with marked sites where EDX analyses were performed; (**c**) micrograph of area E2 with marked detail E3 and sites where EDX analyses were performed; and (**d**) micrograph of detail E3 and marked EDX analysis sites.

During thermal cycling, radial cracks grow perpendicular to the cooled surface. Due to oxidation of the crack canal, the area between cracks also starts to oxidize. Progression of oxidation eventually leads to lateral interconnection of radial cracks. The distance between the early oxidized regions and the cooled surface is about 100–200 µm, with radial cracks are present on both sides (see Figure 7a). Thus, oxidation of several small regions located near each other would lead to their interconnection and consequent early spalling of particles from the cooled surface layer.

EDX analysis was used to reveal the qualitative distribution of chemical elements in the areas of interest, such as highly cracked or oxidized areas in the matrix. The results of the EDX analysis show that areas with reduced Mo content in the carbides exhibit significantly increased carbide cracking. This leads to an accelerated oxidation progression of such eutectic areas, both in the eutectic carbides and in the matrix. Figure 7a (see also details E1 and E2) shows an early oxidation (after 200 cycles at a test temperature of 700 °C) of several separate small eutectic regions arranged in sequence in the lateral direction. Progression of oxidation would eventually lead to their lateral interconnection. The distance between the early oxidized regions and the cooled surface is in the range of 100–200 µm, and radially oriented cracks can also be observed on both sides. Thus, oxidation of several small regions found near each other would lead to their interconnection and consequent early spalling of particles from the cooled surface layer. Details of the two areas marked in Figure 7a are shown in Figure 7b,c, with increased crack density of the eutectic carbides and progressive oxidation in these areas. Formation of oxidation loops (Figure 7b) can be observed in the micrographs of all details. The results of EDX analysis for the sites marked in Figure 7b show reduced Mo content in the carbides (measured between 2.11 and 2.39 wt% in the eutectic carbides and 0.53 wt% in the matrix. Similar analysis of other eutectic carbides showed that cracks occur to a lesser extent (Figure 7d, with detail E4 in Figure 7c and marked locations for EDX analysis) compared to Figure 7b,c, where EDX analysis showed slightly increased Mo content in the range of 2.3–2.9 wt%.

#### 3.4.5. Lateral Crack Branching and Growth Due to Mechanical Bending Stresses

**Figure 8:**
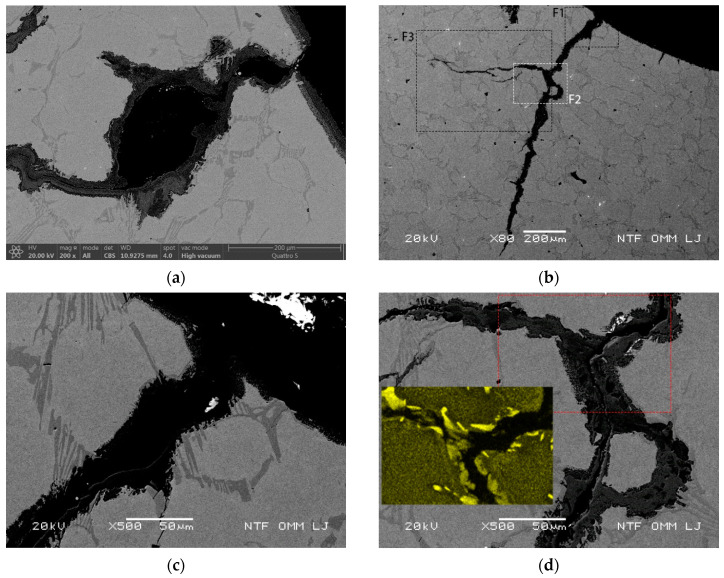

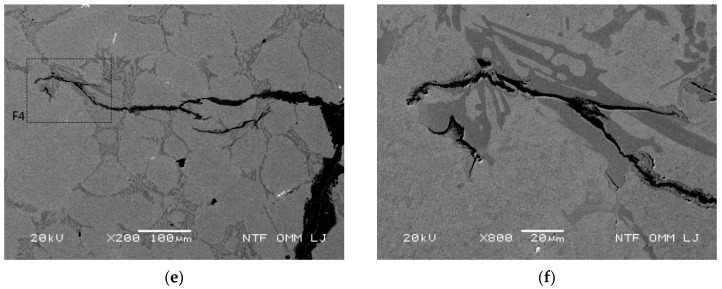
Increased eutectic oxidation-assisted bending stress at a temperature of 700 °C after 2500 thermal cycles. (**a**) High material displacement on the right side of the crack; (**b**) primary and eutectic carbides in successive arrangement and crack growth in the lateral direction due to mechanical bending in the inner area of the specimen with marked detail regions F1, F2 and F3; (**c**) area of detail F1 with oxidation on the cooled surface (specimen bore surface); (**d**) detail area F2, with the initial part of lateral crack growth with the marked area of XRD mapping of Cr; (**e**) detail area F3 with oblique crack growth using matrix and carbide pathways and marked area of detail F4; and (**f**) detail area F4 with crack tip and crack branching.

The process of the eutectic carbide oxidation also occurs behind the crack tip, i.e., the expansion of oxidation in the crack channel, causing additionally increased stresses in the region around the crack tip (increased crack growth), as well as bending stresses in deeper regions lying below the cooled surface and oriented in the lateral direction. Further oxidation of the internal cracks leads to elastic deformations in the boundary layer regions, as shown in Figure 8a, as well as bending stresses in deeper regions. In addition to the formation of cracks in carbide, this can also lead to crack growth that is not oriented in the vertical direction, i.e., crack branching and growth that is also oriented in the lateral or near-lateral direction. Moreover, increased stresses around oxidized eutectics allow for crack growth in other (lateral) directions for the eutectics and primary carbides oriented in the direction of successive eutectics.

An oxidation length of the crack of about 1600 µm is visible in the radial cross section of the specimen (see Figure 8b with the marked points for details F1, F2 and F3), whereas the thickness of the oxidized eutectic on the cooled surface is in the range of 40–45 µm with a length of about 450 µm (see detail F1 in Figure 8c). The oxidation of the initial part of the inner crack was gradual, with consequent low levels of bending stresses inside the specimen. However, the increase in the volume of the material due to the oxidation of the eutectic with a length of about 450 µm from the cooled surface led to an increase in the mechanical stresses in this region. The bending stresses in regions deeper from the cooled surface resulted in lateral crack growth, as shown in Figure 8d,e (detail areas F2 and F3). Detail F2 in Figure 8d shows that the additional growth crack in the lateral direction is related to both the so-called bending stresses and the eutectics, as well as their continuation in the lateral direction and their oxidation. Figure 8e and detail area F4 shown in Figure 8f evidence that crack growth due to mechanical stresses (bending) takes not only the carbide pathway but also grows through the matrix in the lateral direction. Bombac et al. [3] showed that under reduced thermal stresses, crack formation in carbides is reduced, and therefore, crack growth not only takes carbide pathways but also grows in the matrix in the direction perpendicular to the maximum stresses, i.e., the sum of mechanical (bending) and thermal stresses, due to preferential oxidation of the matrix. Furthermore, lateral crack growth can lead to continued lateral or radial cracking, resulting in spalling of the material.

#### 3.4.6. Oxidation in Regions with High Density of Larger Eutectic and Primary Carbides Arranged in a Continuous and Successive Manner in the Radial Direction

Increased oxidation occurs in areas that have a high density of sequentially arranged eutectics and primary carbides in the radial and lateral directions that are connected to the cooled surface. This combination leads to oxidation of extended areas in both directions. Due to increased lateral thermal stresses, the oxidation progress is increased in the radial direction relative to the lateral direction.

**Figure 9:**
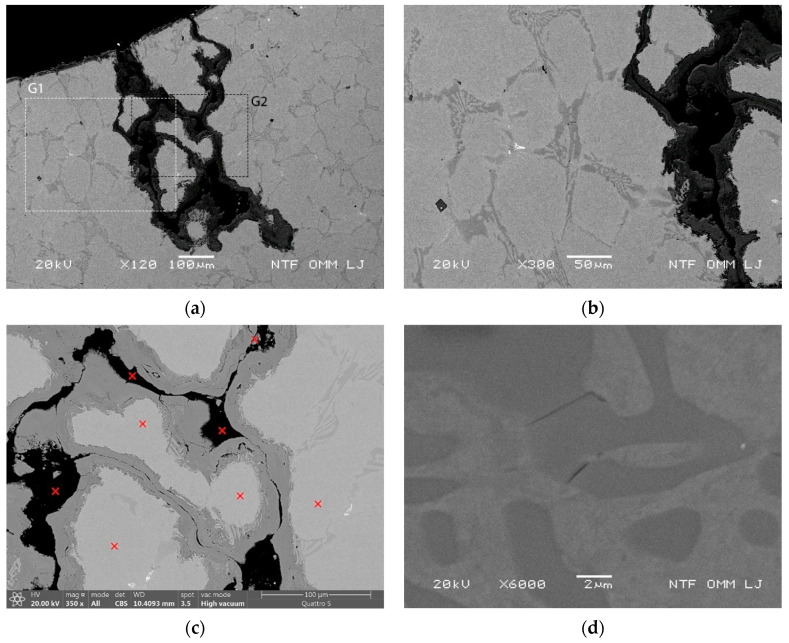
Radial cracks following the path of larger eutectic carbides arranged in a continuous and successive manner tested at 700 °C after 2500 thermal cycles. (**a**) Enlarged oxidation area in the radial direction with marked detail areas G1 and G2; (**b**) detail area G1 with high density of interconnected eutectic carbides; (**c**) detail area G2 with marked positions of EDX analysis; and (**d**) cracks in eutectic carbides near the oxidation tongue.

An example of increased oxidation of areas with high carbide density is shown in Figure 9a, with the highlighted areas of details G1 and G2. Figure 9b shows detail G1 with a high density of the enlarged eutectic assembly and its oxidation. In addition, the EDX analysis showed lower Mo content in the carbides (about 1.87 wt%), as well as in the matrix (0.75 wt%) in a region (see detail G2 in Figure 9c) where oxidation was accelerated (see points of EDX analysis marked in Figure 9c). Detail G3 in Figure 9d shows cracking of carbides, which accelerates oxidation and leads to enlarged oxidation regions. Interconnected eutectics with predominantly large size are thus the basis for oxidation of enlarged regions and, in this case, also for reduced Cr content in the eutectic regions and the narrow band adjacent to the carbides. Furthermore, Figure 9a shows that oxidation extends in the direction of the maximum thermal stresses perpendicular to the cooled surface, causing additional mechanical stresses (bending stresses) in the deeper regions of the sample, as shown in Figure 8.

### 3.5. Identified Mechanisms Leading to Early Spalling of Larger Particles

The following factors are important for the occurrence of early surface degradation due to spalling of larger particles (over 300 μm): sufficiently high maximum temperatures in the thermal cycle with high thermal stresses, strong oxidation conditions, large volume fraction and arrangement of primary and eutectic carbides. The successive arrangement of eutectic carbides in the radial and lateral directions determines the direction of thermal fatigue crack growth. In addition, the cracking of carbides accelerates the oxidation of the primary and eutectic carbides. Oxidation initially occurs in a narrow band adjacent to the primary and eutectic carbides. Oxidation leads to an increase in the volume of oxidized material and thus to an increase in the stresses around the oxidized regions, which is a driving force for lateral crack growth, leading to coalescence of the crack with the adjacent crack growing in the radial direction and resulting in early spalling of the surface material.

**Figure 10:**
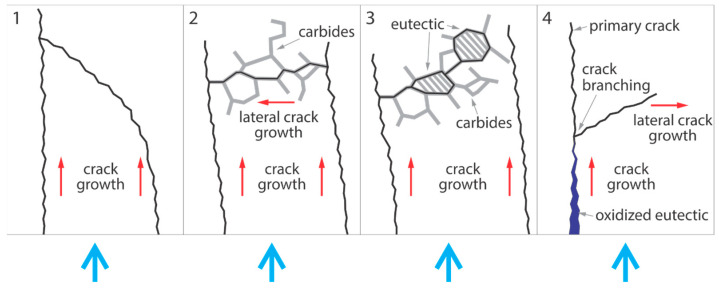
Schematic representation of the four identified mechanisms of crack growth and subsequent coalescence of cracks, leading to early spalling of larger areas. The blue arrows indicate the water-cooled surface, whereas the direction of crack growth is indicated by the red arrows.

The microstructural observations of thermally fatigued specimens and developed surface damages due to distinct types of crack growth leads to their subsequent coalescence and oxidation. This behavior leads to early spalling of large particles, for which several mechanisms have been identified, as described below:

- Direct connection of two radial cracks with enhanced growth starting from the cooled surface, where at least one of the cracks involved does not grow in the vertical direction, i.e., uses carbides, the successive arrangement of which is not perpendicular to the cooled surface, and the connection of which occurs in deeper regions below the cooled surface. This is shown schematically in Figure 10 and labelled 1.
- The crack growth of at least one coalescing radial crack either grows simultaneously in the radial and lateral directions, or the crack changes its growth direction from the radial to the lateral direction, resulting in a connection with an adjacent radial crack. This is shown schematically in Figure 10 in the labelled area 2.
- Crack grows through oxidized regions with small eutectic carbides: initial cracks form near carbides in a eutectic cluster, where the carbide arrangement in the cluster has a higher density and the carbides follow each other continuously. This is shown schematically in area 3 in Figure 10.
- Primary crack growth is followed by extensive oxidation, with crack branching and growth of secondary cracks in the lateral direction at the end of the oxidized area. Lateral crack growth occurs due to oxidation of the thick eutectic, causing bending stresses emanating deeper from the cooled surface. This is shown schematically in area 4 in Figure 10.
- Various combinations of the mechanisms described above.

## 4. Conclusions

In order to investigate the mechanisms leading to premature spalling of large surface particles from the surface of high-Cr steel hot rolling working rolls, thermal fatigue tests were performed on a Gleeble 1500D thermomechanical simulator with maximum thermal cycle temperatures of 500, 600 and 700 °C. Thermal fatigue tests were interrupted after 200, 500, 1000, 2500 and 4500 thermal cycles, and the fatigued specimens were axially and radially sectioned for analysis of the thermally fatigued microstructure and developed cracks. The following conclusions can be drawn from the analysis:

- High test temperatures (600–700 °C) lead to high thermal stresses, which cause cracks in the carbides, as well as accelerated oxidation. Both are crucial for severe surface deterioration.
- The initial microstructure influences oxidation, especially in the presence of eutectic and primary carbides. Carbide cracking accelerates oxidation, and oxidation causes additional stresses due to the increase in volume of the oxidized material.
- The matrix in a narrow band around the primary and eutectic carbides is poor in Cr and therefore more susceptible to oxidation.
- The Mo content in the matrix and carbides varies due to chemical segregations. The higher the Mo content in the carbides, the lower the oxidation rate of the carbides and vice versa. The same applies to the matrix. The brittleness of the carbides decreases with increasing Mo content, and consequently, due to the reduced number of cracks in the carbides, their oxidation rate also decreases.
- Spalling of the surface of smaller particles (100–300 μm) occurs due to the formation of cracks in the carbides and the coalescence of small internal cracks with external cracks.
- Surface spalling of larger particles (above 300 μm) occurs due to a combination of thermal stresses, crack growth, microstructural properties (especially eutectic and primary carbides) and oxidation.
- Four mechanisms of crack growth were identified, and their connection, leading to spalling of larger particles.
- Radial cracks connecting with lateral cracks are particularly detrimental.
- Crack growth also occurs in the lateral direction, with the carbides arranged in a continuous and successive manner.
- Areas with large eutectic carbide assemblies and a high density of eutectic carbides near the cooled surface are particularly prone to oxidation and spalling of larger particles.
- The crack growth rate in the radial direction decreases with increasing depth from the cooled surface, promoting oxidation as the driving force determining the crack growth direction.

## Figures and Tables

**Table 1:** Chemical composition of high-Cr steel in wt%.

C	Si	Mn	P	S	Cr	Ni	Mo	V	Co
1.65	0.66	0.73	0.017	0.009	11.28	1.94	1.17	0.26	0.02

## Data Availability

Data supporting this research may be obtained by enquiry to omm@ntf.uni-lj.si.
